# Learning in emergencies contexts: From the building of the concept to multifaced actions in the field

**DOI:** 10.1007/s11125-023-09639-8

**Published:** 2023-05-03

**Authors:** Abdeljalil Akkari, Myriam Radhouane

**Affiliations:** Faculty of Psychology and Educational Sciences, University of Geneva, Bd du Pont-d′Arve 40, 1211 Geneva, Switzerland

**Keywords:** Refugees, Learning, International organization, Education in emergencies (EiE)

## Abstract

The article is structured into five parts. The first part explains the concept of education in emergencies (EiE) and highlights the challenge of applying it in countries with a fragile education system, particularly in Africa. The second part emphasizes the humanitarian foundation of EiE and recognizes the efforts of international organizations and United Nations agencies in promoting and developing the sector. The third part discusses the quality aspects of EiE, while the fourth part explores curricular choices and potential innovations. Collaboration between national authorities and international organizations is essential for progress in the field, and the choice of language of instruction can be a contentious issue. Finally, the fifth part briefly summarizes the various contributions to the special issue and draws some concluding remarks.

Although a large part of the world was only confronted for the first time with an educational emergency at the beginning of the Covid-19 pandemic, this was not the case for numerous children and young people in Africa, Asia, and Latin America. For them, conflicts and political, environmental, or economic crises had already prevented them from returning to their school benches or curtailed their opportunities for learning. In truth, for these children, the pandemic just made worse a situation that already threatened education.

In fact, access to education for children considered refugees is still not guaranteed: 77% of primary-school-age refugee children have access to school, but only 31% of secondary-school-age children and 3% of students in the higher-education-age-group have access to school (UNHCR, 2020). These figures indicate that thousands of children still do not receive primary education, and the Covid-19 pandemic has only widened the gap for schooling between refugee and non-refugee students. By way of illustration, the Malala Fund estimated that one refugee girl out of two who has left the school due to the lockdowns will probably not return.

In sampling the number of refugee girls attending secondary school in 10 countries that have quality gender disaggregated data (Cameroon, Chad, Ethiopia, Iraq, Kenya, Malaysia, Pakistan, Rwanda, Türkiye, and Uganda), we estimate that half of all girls will not return when classrooms reopen. (Nyamweya, 2020, para. 6)

To diminish the impact of these emergency situations on students’ school careers, education in emergency (EiE) aims for three main objectives: (a) protection, (b) continuity, and (c) reconstruction (INEE, 2004, 2010). These three objectives are interdependent and allow work to progress as much on the educational infrastructure as on the student’s psychological support and teacher training. They demonstrate the wide range of EiE action, its possibilities, but also its complexity.

The first part of this article describes the emergence of the concept and its definition. The second part reveals EiE’s humanitarian foundations. The third part focuses on the quality aspects of EiE. The fourth part discusses curricular choices and future potential innovations. The fifth part discusses each contribution to this special issue of *Prospects*.

## EiE: Recognizing and defining a mobile concept

For several decades, the international community has recognized the importance of providing educational opportunities to all children, whether they are the victims of armed conflict, natural disasters, or other emergency and crisis situations. Therefore, the concept of EiE has gradually emerged as a sector for humanitarian aid as well as for development cooperation. It therefore mitigates the marginalization sometimes observed with education in crisis situations (Taftaf & Williams, 2019).

Through various initiatives—such as the creation of INEE Minimum Standards (Bromley & Andina, 2010) or the inclusion of education in conflict zones as one of the priorities of the International Agenda 2030 (UNESCO et al., 2015)—the international legitimacy of this new field has grown in importance over recent years. The emergence as well as the recognition of EiE have not diminished the complexity of this concept, which is defined, understood, and employed in a very heterogenous manner by both researchers and those involved with education in the field. Its definition requires, in the first place, situating it on a continuum “involving disaster preparedness before a crisis and response in an emergency, extending into early recovery” (INEE, 2004, 2010). This allows the actions undertaken concerning the emergency to be located at different levels, and especially on different time scales. On this subject, the first priority is to welcome the learners as quickly as possible into a place where they will find safety and protection—hence, the importance given to admission and to basic education in the period immediately following the commencement of the emergency.

In these situations, education becomes a true lifejacket: “Educational facilities … can offer children a sense of normalcy, stability, protection, and emotional support, in addition to opportunities to gain critical knowledge and skills” (UNICEF, 2017, as cited in Taftaf & Williams, 2019, pp. 5–6). Indeed, schools have the power to provide a stable environment that helps to overcome trauma; they can provide protection from physical dangers threatening children, particularly ill treatment, exploitation, or recruitment into the armed forces. Frequently, the school gives children access to other vital resources, such as water, food, hygiene, and health care. It is therefore understood that EiE goes beyond the immediate task of transmitting knowledge and functions in close partnership with numerous other fields of humanitarian intervention. The interaction of these different fields increases the effectiveness of educational activities.

In summary, EiE situations can lead to the provision of education and physical, psycho-social, and intellectual welfare, allowing the maintenance of hope for a better life for millions of students (INEE, 2012; Save the Children, 2007).

Naming this field *education in emergencies* is certainly one of the factors that most hinder its implementation since it implies the immediate provision of the actions envisaged. A quick response to a particular emergency would seem to involve short-term measures, whereas the experiences of refugee populations may continue for long periods of time: “Yet, the education in emergencies framework is also imperfect, particularly in its use of the term emergency, which implies a temporary condition and seems ill-suited to describe crises that endure over time” (Burde et al., 2016, p. 623).

Moreover, the terms *education in crises* and *education in protracted crises* are sometimes employed to “clarify the connection to emergency while preserving the sense of urgency intended to garner a response” (Burde et al., 2016, p. 623). These authors explained that they continue to use the term *education in emergencies* since it corresponds to the phrase employed by the majority of people working in this domain.

Table 1 summarizes the different terms employed in the analysis of EiE. The complexity raised by the definition of EiE shows, nevertheless, the difficulty of conceiving a school enrollment, as well as an education, able of confronting the future in a context that is unpredictable and mobile, by definition.

**Table 1:** The range of EiE situations

	Nature of the crisis	Main challenges	Educational issues
Children and young refugees	Political oppression or armed conflict (including acts of terrorism) leading to an important population shift and the crossing of an international frontier	Planning the provision of education in a context of continuing uncertainty Development of educational supply with limited resources (structural, human, and financial) Psychological, social, and emotional impact of the crisis	Choice of the teaching language Choice of the educational system (national or parallel) Recruitment of qualified teachers Student support (developing resilience)
Internal displacement	Political oppression or armed conflict (including acts of terrorism) leading to an important population shift within a country	Planning the provision of education in a context of continuing uncertainty Development of educational supply with limited resources (structural, human, and financial) Psychological, social, and emotional impact of the crisis	Adaptation of the national educational provisions In some cases, choice of the teaching language Recruitment of qualified teachers Student support (developing resilience)
Children and young people victims of natural disasters	Destruction of educational infrastructures and the regular circumstances of life	Rapid reconstruction of the educational system and infrastructures Development of educational supply with limited resources (structural, human, and financial) Psychological, social, and emotional impact of the crisis	Continuity of educational provisions Support for the students and educational personnel (developing resilience) Preparation for future crises
Children and young people in a health crisis (e.g., the Covid-19 pandemic).	Impossibility to attend school	Implementation of distance teaching and weak technological infrastructure Planning the provision of education in a context of uncertainty	Continuity of educational provisions Support for the students and educational personnel (developing resilience)

*Source*: The authors

## EiE: A field rooted in humanitarian approaches

EiE is not a field without its own history and origins. As mentioned by Save the Children (2021b), numerous approaches provide it with its structure and have encouraged its development, as well as its implementation. This organization referred particularly to the *humanitarian principles,* the *humanitarian code of conduct*, the *sustainable development goal 4* (SDG4), or the *INEE minimum standards.* In this section, we will discuss these approaches in order to understand the extent to which they influence the field of EiE.

Humanitarian principles are an initial way of understanding the context of EiE: they can guide the actions of different participants, while supporting the development of educational projects, encouraging interactions with vulnerable populations, or even enabling the analysis and taking stock of the actions undertaken. The four principles as well as their definitions are:

- *The principle of humanity*: This principle means “that human suffering must be addressed wherever it is found, with particular attention to the most vulnerable” (European Commission, 2020).
- *The principle of neutrality*: “Humanitarian actors must not take sides in hostilities or engage in controversies of a political, racial, religious or ideological nature” (UNOCHA, 2012).
- *The principle of impartiality*: “Humanitarian action must be carried out on the basis of need alone, giving priority to the most urgent cases of distress and making no distinctions on the basis of nationality, race, gender, religious belief, class or political opinions” (UNOCHA, 2012).
- *The principle of independence*: “Humanitarian action must be autonomous from the political, economic, military or other objectives that actors may hold with regards to areas where humanitarian action is being implemented” (UNOCHA, 2012).

These four principles allow one to grasp humanitarian action, the place occupied by its activities, its relationship with local communities and with refugee populations, etc. Their usage also allows light to be shed on the tensions those involved with humanitarian projects may experience. In their study, Broussard et al. (2019), for example, drew attention to tensions between commitment in the field and the principle of neutrality. Their work allows us to stress the complexity of implementing principles in the politically unstable situations often targeted by EiE.

Otherwise, it is important to employ a certain flexibility in the understanding and introduction of these principles: “Far from being rigid and dogmatic, these principles can bend to fit the context, the forces at work and the sensitivities of the various groups” (Labbé & Daudin, 2015, p. 188).

The Code of Humanitarian Conduct, signed by 492 organizations (UNOCHA, 2012), is another resource contributing to the humanitarian approach. It consists of ten principles pleading for, among other things, the culture of the beneficiaries of humanitarian actions to be taken into account (principle 5), the involvement of local communities (principles 6 and 7), and support in a crisis but also the anticipation of future situations (principle 8). Here again, the context allows support and questioning of the development of EiE in order to contribute to maintaining its relevance for the populations it is intended to serve.

The third approach mentioned by Save the Children is SDG4 (quality education). This objective of sustainable development is broken down into different targets that can both guide EiE activities and also encourage and support their development. These targets concern, for example, access to preschool education (Target 4.2); the elimination of sexual inequality (Target 4.5); and the strengthening of access to all levels of education, including those of vocational and university education (Targets 4.3 and 4.4; United Nations, 2020).

These objectives correspond to EiE situations to the extent that access to quality education should be ensured for all learners, whether they come from a country in the North or the South, whether or not they come from a conflict zone, whether they live in a refugee camp or in their own country, etc. EiE situations are designed to overcome the various obstacles preventing the achievement of the right to education. It should, however, be stated that some projects falling within SDG4 do not appear to form part of the context of sectorial education plans (i.e., the national planning of educational projects); from this observation, the result could be a poor connection between national institutional approaches and reality in the field. In other words, while parallel projects are being developed, opportunities may be missed for collaboration and for the integration of education for refugee children into national education strategies.

Finally, the fourth approach we will mention here is the one prepared by the INEE network. It is entitled *INEE minimum standards* and forms part of a list of criteria situating the educational approach in emergency situations. These standards are the outcome of a wide-ranging collaboration (INEE, 2004, 2010), which has allowed the challenges of relevance, as well as their pertinence to EiE issues, to be pinpointed. Nevertheless, one can observe a real conflict between the collaborative approach designed to accommodate local concerns and regulating the field involving these standards (Bromley & Andina, 2010).

The objective of the minimum standards is “ensuring quality, coordinated humanitarian response: meeting the educational rights and needs of people affected by disaster through processes that assert their dignity” (INEE, 2004–2010). INEE organizes its standards into three main groups (community participation, coordination, analysis), which represent the foundation for the development of other standards. These are then divided into four categories (allowing for the identification of twelve standards altogether).

- *Access and learning environment*: The three standards falling into this category intend to promote equal access, the learners’ social and physical well-being, as well as the security of the learning environments.
- *Teaching and learning*: The four standards in this category focus on teaching content, learning and teaching strategies, teacher training, as well as the evaluation of learning outcomes.
- *Teachers and other education personal*: The three standards in this category are concerned with recruitment, and the training and support (or supervision) provided for teachers.
- *Education policy*: The two standards in this category are concerned with the legislative and political aspects associated with the implementation of the educational project in an emergency situation. (INEE, 2004-2010)

Even though the first INEE publication dated from the early 2000s, it seems that the standards are not yet fixed; on the contrary, they are open and adaptable to the evolution of EiE. For example, the way in which the 2010 standards dealt with the concept of resilience demonstrates their capacity for evolution and adaptation. Indeed, while at their inception, the standards did not mention resilience, today it is a key concept that also forms part of the glossary included in the framework document defining each standard (Shah et al., 2019).

The humanitarian approaches presented here allow EiE to be placed on firm, internationally accepted foundations, standardized to encourage collaboration but sufficiently flexible to enable them to be adapted to the evolution of local reality. These approaches support action and make it possible to be more relevant, inclusive, measurable, and coordinated in order to satisfy the obligation of schooling represented by access to education for thousands of children without hope.

Finally, even though the value of these approaches has been established, we can still conduct a critical appraisal of them. In the manner of Bromley and Andina (2010), it is important to question the bias resulting from the process of standardization of a field as complex and fragile as EiE. We can also query the place of national governments in the supra-national frames of reference and, in this way, the balance of the relationship between institutional actors.

In summary, these approaches provide a theoretical foundation when reflecting on educational action in emergency situations, a frame of reference for evaluation, and a structure for cooperation, but they do not mean that a contextual analysis and critical appraisal of each situation can be avoided.

## Quality, relevance, ownership, and empowerment

Once the problem of access to basic EiE situations is resolved, attention turns to matters of quality. Indeed, various research studies have brought to light the limited learning acquired by children receiving EiE. Their early departure from the school and other difficulties have been well documented (Burde et al., 2017; Karam et al., 2017; Shohel, 2020).

In the first place, the idea of quality entails turning our attention to the implementation systems in the context of educating refugee students. While in northern countries, most students are enrolled in national educational systems, in the Global South, two alternatives exist. The first consists of the rapid introduction of educational structures within refugee camps. This makes it easier to choose the teaching language, due to the relative cultural homogeneity of the people within the camp. Nevertheless, these situations resemble open-air prisons in which the most basic resources are generally unavailable. Furthermore, the Covid-19 crisis resulted in a significant reduction in the staff of nongovernmental organizations (NGOs) in some refugee camps—hence the necessity for an increased inclusion of refugees as the main actors in their education. The second option concerns refugee children who enter national educational systems. This system has been employed in the case of Syrian refugee children in Jordan and in Lebanon, and for the same type of student in Türkiye (Hos, 2016). This solution has the advantage of benefiting from existing infrastructure to provide education for refugee children, frequently in the latter part of the day. Other children, though less numerous, have been welcomed in public schools alongside local children, which has of course resulted in overcrowded classes and sometimes tensions between refugees and national citizens. In these contexts, the question of the choice of teaching content has also been raised.

In order to react to the various issues concerning the quality of educational provision, several innovations have been introduced. For example, the town of Gaziantep (a Turkish city near the Syrian border) opened a community school enrolling refugee students and following the Syrian curriculum. While this school is an interesting initiative, its development raises various problems concerning the quality of education, such as the lack of socio-emotional support (for both the students and the teachers), the recognition of the school (and therefore the education received by the students) by the Turkish authorities, and the number of students per class (Hos, 2016). This study draws attention to the complexity of providing quality education in a situation affected by emergency and crisis.

The question of the quality of educational undertakings is of major importance if, on the one hand, the right to education is to be achieved, and on the other, if it is to be a source of emancipation, autonomy, dignity, and so on for refugee populations. UNESCO (2019), through its political approach and the analysis of refugee education, provides a relevant theoretical framework.

This approach is based on the “four As”: accessibility, availability, acceptability, and adaptability. *Accessibility* emphasizes that it is essential for the refugees’ right to education to be guaranteed by legislation and through the national political framework for education. The educational system in the host country, its institutions, and its curricula must be accessible for everyone at all levels of education, by guaranteeing information, non-discrimination, and physical and economic accessibility. *Availability* means the inclusion of refugees within national educational systems. Infrastructures should be properly equipped, and the teachers suitably trained, qualified, motivated, and provided with adequate salaries. Nevertheless, in the field, accessibility and availability are not always respected. As illustrated by the study of Hos (2016), numerous refugee students in the Global South receive their schooling in structures parallel to the national educational system. *Acceptability* refers to the need for the form and content of education (including the curriculum) to be relevant and culturally appropriate for all refugee students. Education for human rights, respect for cultural diversity, intercultural understanding, and multicultural education can then play a significant role in emergency situations. On this subject, the teaching language can represent a major obstacle for refugee education. From the primary to the higher education level, access to educational content can be obstructed (or at least made more difficult) if students are asked to adapt to a foreign language. Finally, *adaptability* means that education should be flexible in order to conform to the refugees’ constantly changing needs.

The context of the four As brings together the dimensions of recognition, redistribution, representation, and reconciliation to explore what could look like the consolidation of sustainable peace seen from the perspective of social justice (see Fraser & Halpbern, 2013). It is vital that the cultural translation of these concepts become locally integrated interpretations (Novelli et al., 2017). It would then seem that this approach could be a considerable resource to combine the necessity of access with that of quality in the EiE context.

## Curriculum, educational alternatives, and potential innovations

As we have mentioned, the nation-state remains the agency that fixes the priorities and directions of education through national strategies reflecting international agendas. From this point of view, over recent years, sectoral education plans have become the principal tool in the planning of education in the countries of the South. These plans grant access to some international financing, such as, for example, the Global Partnership for Education (GPE). However, sectoral plans that incorporate some part of EiE are rare, even in those countries of the South most concerned about this problem; this is the case despite the commitment of the GPE to EiE (Menash & Dryden-Peterson, 2015). The question of educational planning, including the sector’s strategic choices, in emergency situations is thus raised. Lastly, it is our intention to tackle two difficult aspects of this matter: the choice of the teaching language and curricular guidelines. We will then deal with educational alternatives, as well as the possibilities for innovation offered by the field of EiE.

### The choice of the teaching language

Teaching in the mother-tongue, at least during the first years of schooling, is the hallowed principle of international organizations as well as of research. Nevertheless, with EiE, this principle is confronted by two major obstacles. First, many parents and learners in emergency situations desire to settle permanently, preferably in a country of the North. For this reason, it is possible they would like to be educated in English. Even so, many wish to return to their country of origin; therefore, the host country’s language is not suitable for students intending to maintain the continuity of their school career. In some cases, the host country seeks to impose its official teaching language, with the intention of both controlling education and offering employment opportunities to local teachers. Indeed, the presence of numerous national and international NGOs in EiE renders it attractive to retired teachers (e.g., in Jordan) or national teachers seeking to earn more.

The teaching language therefore raises questions: Should a language useful for social and economic integration in the host country receive priority? Or should instruction be in the language of origin, with a view to an eventual return? These questions are faced by economic migrants, while contemporary research tends to favor interaction between the language of origin and the teaching/host language. In the case of education for refugee students in the countries of the Global South, their perilous situation as well as the nature of emergency add to the need for reflection and make it more complicated.

### Curricular guidelines

The choice of curricular guidelines also causes problems in the context of EiE. In fact, school curricula are usually constructed on national values and priorities. Different questions can then be asked: Should school courses be based on interculturality or on education for global citizenship? And what curricula should be proposed for refugee children originating from neighboring countries? For example, should Syrian refugee children in Jordan follow the Jordanian or the Syrian national curriculum? For refugees in Niger, who come from Mali, Nigeria, and Burkina Faso, difficult curricular choices are complicated by hesitation about the teaching language.

The difficulty in finding the right answer for the curriculum is closely linked to uncertainty about the future of refugee children and their families; does their future entail a potential return to their country of origin, a long-term precarious existence in the first host country, or admission to a stable northern country? As a way out of this impasse, a multilingual and multicultural curriculum could be an interesting solution. On this subject, Schweisfurth’s (2015) proposals are interesting avenues for strengthening EiE by mixing appropriate teaching practices with a relevant curriculum:

1. Lessons that engage students and motivate them to learn
2. Mutual respect between teachers and learners, and a communal atmosphere and interpersonal relationships that reflect this
3. A curriculum that builds on learners’ existing knowledge and skills
4. The importance of dialogic teaching
5. A curriculum relevant to learners’ present and future lives;
6. A curriculum and pedagogy embracing skills and attitudinal outcomes, as well as the acquisition of knowledge
7. Assessment processes that are meaningful for those being assessed so their learning is improved (pp. 263–264)

### Educational alternatives and innovations

Through its structure and organization, the traditional form of the school is not necessarily the most appropriate solution to respond to the issues of EiE. For this reason, it seems important to us not to neglect the initiatives arising from informal education or other teaching alternatives in order to achieve an increasingly relevant and innovative EiE.

One of the alternatives to formal education that we have chosen to present here is schooling available through information and communication technologies (ICTs). Their use has become one of the priority approaches in EiE (Dahya, 2016; Lewis & Thacker, 2016). This is particularly true for higher education (Milton, 2017; Reinhardt et al., 2018). Numerous universities in the Global North, in fact, offer courses using distance education intensively. This choice is pragmatic in that distance education relieves the teachers and the designers from the need to travel, which has the advantage of reducing security risks. In addition, ICTs can represent an educational innovation by ending learners’ isolation in emergency situations and making access to computerized resources more democratic. Mobile learning then represents a low-cost innovation that introduces ambitious educational and social innovations.

Alongside this list of advantages to the use of ICTs in the field of EiE, studies have drawn attention to the complexity and obstacles encountered in their use as teaching methods. For example, the majority of educational courses have been designed to be viewed on computer screens, whereas refugee students for the most part have access to smartphones (Taftaf & Williams, 2019). This difference in format would seem to reduce access to the teaching content. The weakness of networks has also been described as an obstacle in the use of ICTs (Dridi et al., 2020; Taftaf & Williams, 2019). Finally, the relevance of training, even online, appears to depend on the possibility of local populations participating in their creation (Taftaf & Williams, 2019), as well as on the ability of the trainers to understand the conditions in which the learners are receiving educational content (Dridi et al., 2020).

ICTs represent a major source of innovation for EiE. They allow the lack of infrastructure and the lack of teachers to be overcome and allow for the creation of links between scattered populations, as well as for ICTS participation in the democratization of curricular content. Nevertheless, their introduction necessitates reflecting about the factors that may hinder their use. The introduction of ICTs illustrates the complexity of EiE and the importance of ensuring the suitability of projects to the issues facing them, as well as evaluating projects in order to improve them.

Finally, for these innovations to work, it is important to recall the indispensable role of teachers. Whether they are themselves refugees or not, teachers provide the guarantee of quality and relevant EiE. In general, we lack information about the profiles of teachers working in emergency situations. Moreover, research tends to stress the paucity of pre-service and in-service training.

## Presentation of the contributions and concluding remarks

The contributions in this special issue of *Prospects* come from diverse institutions and regions worldwide, reflecting the perspectives of both researchers and actors in international organizations. Together, they highlight the breadth and complexity of the field of EiE.

Among the five contributions focusing on emergency contexts, Mickaël Idrac examines the schooling of refugee children in camps in Greece, where restrictive migration policies and educational system challenges create unique educational complexities.

Albane Buriel analyzes the education system implemented by the Islamic State in Iraq and Syria, shedding light on the totalitarian educational tools used by the group.

Rola Koubeissy, Geneviève Audet, Garine Papazian-Zohrabian, and Olivier Arvisais explore the experiences of teachers working with Syrian refugee students in Lebanon, emphasizing the importance of support for teachers in ensuring the well-being and safety of refugee students.

Mohamed Sagayar Moussa examines the education conditions for refugee and internally displaced children in Niger, where an already weak education system is further challenged by high dropout and absenteeism rates among students and teachers.

Finally, M. Mahruf C. Shohel analyzes the Rohingya crisis from a human rights perspective, highlighting the traumatic experiences of Rohingya children and the challenges they face as refugees in Bangladesh, particularly during the Covid-19 pandemic.

Overall, these contributions provide valuable insights into crisis situations and the perspectives of key stakeholders, contributing to a better understanding of the development and objectives of EiE.

The following four articles present solutions that have been developed to address the needs of EiE:

Pilar Aguilar and Petra Heusser analyze the emergence and launch of the Global Hub for Education in Emergencies in Geneva, which aims to mobilize funding, efforts, and pedagogical action for the education of the most vulnerable children worldwide.

Martin Johnson, Sinéad Fitzsimons, and Victoria Coleman address the challenging issue of designing curriculum for EiE, recognizing it as an “ill-defined problem space” for curriculum developers. They propose a 3-step curriculum framework as a useful design tool and stress the importance of considering the nature of knowledge in skills development.

Paul O’Keeffe and Thibault Lovey compare and evaluate a medical studies course delivered via blended learning in the Kakuma refugee camp, which was contextualized to the camp's specific needs, with a non-contextualized version of the same course delivered in the Dadaab refugee camp in 2018. Their study finds that the contextualized course achieved better learning outcomes than the non-contextualized version.

Olivier Arvisais, Patrick Charland, François Audet, and Yannick Skelling-Desmeules analyze the protection and sense of safety of students enrolled in an accelerated education program in the Dadaab refugee camp. The purpose of their article is to address a gap in the literature on violence against students in humanitarian crisis situations. It does this by comparing the risk perceptions of young refugees when they were out of school to when they were enrolled in an educational program, aiming to challenge the widespread assumption that education protects children. The article argues that this assumption is not always accurate and that the reality is much more complex. The study utilized a mixed method approach with a sequential exploratory design that prioritizes data, lived experience, and fieldwork. The article presents a case study of the accelerated education program in the Dadaab refugee camp. The findings indicate that while enrolling in school may reduce some risks, it can also increase others, such as physical assault and gender-based violence.

Finally, Myriam Radhouane provides a comprehensive and structured literature review on the education of refugee students or those in the asylum process in the Global North. Given the complexity of the migration journey, she examines 50 studies to document how schools in the Global North organize and address the education of these students and what are the primary pedagogical challenges. Radhouane identifies a common tension between the urgency of providing education and the need to analyze school environments.

As a conclusion, it is important to acknowledge that although the field of EiE is well-established in the humanitarian domain and international development cooperation activities, it remains an emerging field for educational research worldwide. Therefore, it is crucial to encourage its consolidation through empirical, comparative, and international research. Reflecting on the underlying theoretical approaches is also essential, including understanding the extent to which these approaches fit into different categories, such as emancipatory, participatory, or liberal.

Encouraging the development of empirical research in the field of EiE is justified by the lack of reliable information, but also by the variable nature of emergency situations. Some situations are ongoing, new ones appear, and all of them have a high degree of instability. In addition, research should grant access to the life stories of students and the subjective experiences of other actors in EiE situations (e.g., project leaders, teachers, parents)—all of which have an impact on the educational outcomes of academic learning as well as competencies, critical spirit, emancipation, and more. It also seems important to encourage longitudinal research to better understand the processes affecting a student living in an emergency or crisis situation. This research format allows for the examination of how interventions using EiE influence a school suffering from crises, changes of school location, and disruptions in lessons.

Other methodological approaches would allow for the following analyses: *participative* approaches would enable greater emphasis on daily reality, opinions, and the experiences of the population targeted by EiE or those actors concerned by its implementation; *quantitative* approaches would permit the collection and updating of reliable data concerning the schooling of students in emergency situations. These approaches would allow projects in the field of EiE to be founded on the outcomes of recent research, while reflecting what is taking place in the field. *Comparative* approaches would allow solutions to be tried out in different contexts and, in this way, to identify strengths, weaknesses, and opportunities.

It is important for comparative and international analyses to be inspired by existing structures, such as the INEE minimum standards, in order to (a) guarantee different viewpoints (e.g., the views of local communities involved in the implementation of EiE) and (b) facilitate an international and reproducible comparison. Moreover, this does not prevent the researchers from taking a critical look at the resources. These international comparisons can be carried out for the benefit of refugee students, to the extent that they can thereafter be used as major resources when choosing solutions for different crises and emergency situations. The comparative approach (as well as the distribution of research) also has the benefit of highlighting local innovations and new projects, which have not necessarily been made known on a major scale, but whose relevance could of use to other students.

To make EiE truly international requires increased visibility and participation on the part of researchers and actors from the southern world. At present, they are sometimes present in the context of unequal partnerships but are not yet represented at all levels of EiE. If initiatives take steps in this direction, it is vital to encourage the active participation of all actors.

Lastly, research would permit a critical view of what is needed to support and improve EiE; however, for this to happen, it is necessary to position this research so its results can become a resource for the largest possible audience. This would entail, for example, publication of the results in Open Access, international exchanges between universities in the South and the North, and the further development of participative methodologies.
